# Decolonizing scholarship? Plural onto/epistemologies and the right to science

**DOI:** 10.3389/fsoc.2023.1297747

**Published:** 2023-11-17

**Authors:** Caroline Gatt

**Affiliations:** Institut für Kulturantropologie und Europäische Ethnologie, Universität Graz, Graz, Austria

**Keywords:** decolonizing academia, epistemic colonialism, the pluriversity, the human right to science, onto/epistemology

## Introduction

In recent years there has been a significant increase in the recognition that non-Western epistemologies are legitimate and valuable ways of knowing. In December 2022, for instance, guidelines were issued by the White House Office of Science and Technology which now recognize that “in order to make the best scientific and policy decisions possible, the Federal government should value and, as appropriate, respectfully include Indigenous Knowledge” (Aluaq Daniel et al., 2022). The annoucement of this development cited Cheryl Andrews-Maltais, Chairwoman of the Wampanoag Tribe of Gay Head Aquinnah, who was involved in the Tribal consultations that led to the new guidelines. In these consultations she said:

“Had our traditional cultural practices and ceremony not been outlawed and had our information keepers been listened to over the centuries, we probably would not find ourselves in the position we are today—with the losses and extinction and contamination we face as our global community. This is a valuable component of being able to face not only climate change but the preservation and protection of all of our resources.”

(Cited in Aluaq Daniel et al., 2022)

Heeding Cheryl Andrews-Maltais's words implies taking seriously calls for decolonizing science and scholarship. If it is to improve people's lives, enhance the enjoyment of other rights and contribute toward achieving the SDG goals, any implementation of the right to science will need to address and redress the ongoing epistemic colonialism in the practices of the majority of scientific and scholarly work.

In this opinion piece I offer an outline and analysis of:

- epistemic colonialism;
- decolonizing academia, and its current pitfalls;
- and the notion of the “pluriversity” (Mbembe, 2015).

The goal of these outlines and analyses is to show that what is required in order to intrepret and implement the right to science in the spirit of freedom and self-determination (Article 1 and Article 3) of the Universal Declaration of Human Rights is that in the first place science and scholarship need to cease to perpetuate coloniality. Most of the examples of ongoing epistemic coloniality that I present are drawn from anthropology, where I situate my work. Considering how anthropology purports to be one of the few academic disciplines that introduce different ways of knowing into university contexts (Strang, 2006), it can be inferred that the situation is similar if not much more problematic in other conventional disciplines.

## The human right to science

The human right to science is part of article 27 of the Universal Declaration of Human Rights (1948). Article 27 reads:

1. Everyone has the right freely to participate in the cultural life of the community, to enjoy the arts and to share in scientific advancement and its benefits.
2. Everyone has the right to the protection of the moral and material interests resulting from any scientific, literary or artistic production of which he is the author.

In a recent keynote speech at the “International conference on the Human Right to Science” (2022), Special Rapporteur on cultural Rights, Shaheed, explained how, when she took on her current role, those approaching this article from a cultural rights lens ignored the reference to science, where as those interested in the right to scientific progress considered the inclusion of culture as “coincidental” (Shaheed, 2015). This in itself highlights the relation of Western Science to cultural questions. On the one hand, from a certain scientific perspective, culture and science are not related domains. Latour (2004) has argued that the discourse of “Science” (and not the day-to-day practices of science that go on in labs), portrays the world as mono-natural and multi-cultural. In other words, “Science” and scientists deal with the “real” world, where as “cultures” present multiple, derivative “beliefs” about the world overlain on top of a single nature (as described by Western science). On the other hand, since the history of this “Science” discourse has its roots in Europe, why would cultural rights proponents from outwith Europe, where the need for cultural rights to be upheld is most pronounced, need to promote a heritage that often disparages their own? Indeed, historians and anthropologists are increasingly finding evidence that in this discouse of “Science,” Western scientists have routinely erased the Indigenous contributions in scientific developments (see e.g., Safier, 2010; Gruzinski, 2013; Giraldo Herrera, 2018).

Rather, Shaheed argues that the human right to science and culture *must* be understood as closely related. Further, she suggests a closeness between the human right to science and cultural rights in Article 27 with the right of all peoples to self-determination. She explains that article 27 relates to human creativity, in other words “the human pursuit of knowledge and understanding complemented with creative responses to a constantly changing world.” She goes on, “the ability to aspire, is an important cultural capability for aspirations are never merely individual exercises, they are informed by, and in turn inform, communities of shared cultural values and draw upon cultural heritage including accessible accumulated scientific knowledge.”

Shaheed therefore links the cultural with scientific innovation in a feedback loop. Drawing on and contributing to individual and shared cultural heritage, the drive in scientific endeavors to further understand and better inhabit the world, which in turn leads to further aspirational possibilies. However, in order for the close relationship between science and culture, that Shaheed argues for, to be realized, it is the very understanding of what science and knowledge are that need to be reformulated. As alluded to above, “Science” has participated in the subjugation of different epistemologies, different ways of pursuing knowledge, based on the colonial project that sought, and often still seeks, to portray Western understandings as universal and fundamental, while others are at best “beliefs,” at worst “mistaken” and in need of correction (Escobar, 2018; Robinson, 2020).

## Epistemic coloniality and epistemic violence

Epistemic coloniality was a key tool of European colonial forces of expansion and control. Even with the process of political decolonization in the 20th Century, many forms of coloniality persist, and reproduce and maintain local/global imbalances of power based on colonial lines of inclusion and exclusion. Through the often fiercly protected position that only Western scientific, rational epistemologies generate “proper scientific knowledge,” epistemic colonialism continues to perpetrate epistemic violence on the bearers of other knowledges.

Epistemic coloniality is characterized by a hierarchization of knowledge that privileges “Western” rational academic knowledge, and has been used as the justification for epistemicide and underpins all other forms of imperial expansion and subjugation (Santos, 2018). In this hierarchy human “races” are classified according to the sense organ that supposedly characterizes them. This classification positions the white European “eye-man” at the top and the Black African “skin-man” at the bottom (Lorea, 2022). Carola Lorea shows that this classification is also gendered: “Women were associated with debased oral culture, gossip, folklore, the realm of tales and superstition, while men were assumed to represent the propriety of grammar, logic, and literature” (Lorea, 2022, p. 842).

Here are two examples of how coloniality and racism are justified and underpinned by epistemic coloniality.

In the US, Sun Eidsheim (2018) shows how the pseudo-science of craniometry, in which already determined, and racist, categorizations of humans, was used to justify racist policies that persist today. These assumed human categorizations, already active in the subjugation of slaves in nineteenth century America (Smith, 2001), became established as supposed “scientific fact.”

In Canada, the tragedy of the Residential schools was justified as bringing “civilization” to First Nations people. The residentials schools were part of a wider complex of actions that resulted in genocide and cultural genocide being committed against Indigenous peoples by the Canadian state. In addition to the residential schools, this genocide and cultural genocide was carried out through laws such as the Indian Act, including the “potlatch ban” (1884–1951), and the institution of reserves, to which Indigenous peoples were confined. The residential schools detached children from their traditional sources of learning, and subjected the children to sordid violence and abuse, including prohibiting them from speaking their native languages and honoring their ties to ancestors, spirits and the land (Robinson, 2020, p. 150). Prior to colonial settlement and the oppressive regime that followed, Indigenous families and communities in Northwest Canada would travel widely for different seasonal activities, including harvesting, hunting and fishing, and important gatherings such as potlatches (Robinson, 2020, p. 55). “All of these forms of control over the movement of Indigenous bodies did not just limit mobility, but fundamentally restricted the range, flexibility, and time of attention more generally, by restricting Indigenous *proprioceptive agency* within (and in relation to) [their] lands.” (Robinson, 2020, p. 55, emphasis added). The effect of these restrictions was to forcibly change their ways of perceiving, knowing and studying the world, or in other words this was also epistemicide.

Such epistemic violence continues in daily life for First Nations people. Robinson (2020), xwélmexw (Stó:lo) sound and Indigenous studies scholar, gives a concise example of epistemic violence:

Ontologically, many of our songs have their primary significance as law, history, teachings, or function as forms of *doing*. This is to say they are history, teaching, law that take the form of song, just as Western forms of law and history take the form of writing. Yet they cannot also be reduced to merely an alternative form of Western documentation—the exact equivalent to a book, or to written title of land. I have been repeatedly asked to account for the ways in which our songs serve as law, or how songs have life. At the heart of these questions has been a demand to explain how our songs fulfills the necessary and sufficient *Western* criteria that constitute a thing. To measure the “fit” of Indigenous processes by Western standards subjects them (and the Indigenous person who explains them) to epistemic violence, and reentrenches colonial principles and values.

Robinson (2020, p. 46)

## Epistemic coloniality in academia

Even in a discipline which purports to focus on human difference as a shared good, epistemic coloniality shapes “dominant anthropologies.” “Dominant anthropologies” are those anthropological traditions and disciplinary contexts in colonial metropoles, such as in the US, the UK, France and Germany (Escobar and Restrepo, 2005). In such dominant anthropologies different ways of knowing have mostly been introduced into the university *by stealth* (Pels, 2000), if not by outright appropriation. For instance, where only European scholars are cited rather than the Indigenous scholars or knowledge bearers themselves, this is a form of appropriation (Todd, 2015). To a limited degree, anthropologists have managed to generate increased understanding and respect for subaltern ontologies and epistemologies. However, apart from having an effect mostly limited to discussion in the discipline itself, it is also a rhetoric that assuages guilt and does not call for deeper structural disciplinary change (Gatt, 2022).

By introducing concepts, approaches, pedagogies, epistemological and ontological possibilities from Indigenous peoples and other subaltern knowledges in this “stealth” or appropriative mode, what has mostly happened is a reproduction of the hegemonic hierarchy of knowledges. For example, Anishabee and Haudenosaunee scholar Watts (2013) critiques Donna Haraway's use of the notions of Coyote or the Trickster. On the one hand, Watts appreciates how Haraway's feminist antiessentialism works to undermine universalist depictions of knowledge. However, on the other hand, Haraway uses concepts derived from localized knowledge without taking into account the Indigenous histories and protocols around such knowledge and stories. Watts points out how in this way Haraway promulgates understandings of “knowledge” dictated by Western principles, where Indigenous stories become abstracted, and extracted tools for Westerners' use. Essentially, what this does is “erase the embodied, practiced, and legal-governance aspects of Indigenous ontologies as they are enacted by Indigenous actors” (Todd, 2015, p. 17). Although there is increasing recognition and respect for non-Western, non-hegemonic ways of knowing, epistemic colonialism still characterizes the large majority of scholarship.

## Onto/epistemology

The subtitle of this opinion piece is “Plural onto/epistemologies and the right to science.” It is important to explain what the term onto/epistemology refers to. The term indexes that questions of epistemology are inextricable from ontological ones. The notion that knowledge can be abstracted from the ways of life from which it emerges, is a characteristic of Western Science. Therefore, any discussion which separates epistemology from ontology perpetuates Science's dissociation of knowing from being. The key that links this to coloniality is that through this dissociation Western Science treats the world as furnished by *objects* of knowledge, as if they are ready to be extracted for collection, analysis and future application. In this way Western Science's understanding of “knowledge” parallels exactly with understandings of human and natural resources as extractible and exploitable.

For instance, Native American scholar, Shawn Wilson makes the connection between epistemology, ontology, and methodology. He writes “[l]ike myself, other Indigenous Scholars have in the past tried to use dominant research paradigms... We have tried to include our cultures, traditional protocols and practices into the research process through adapting and adopting suitable methods. The problem with that is we can never really remove the tools from their underlying beliefs” (Wilson, 2008, p. 13). Therefore, scholars will need to recognize that the understanding of knowledge as a free floating abstractable “good” perpetuates epistemic colonialism (Robinson, 2020, p. 158). Indigenous scholars have already offered alternative emplaced and situated understandings of knowledge. With the principle of “Indigenous Place-Thought” Watts (2013) proposes an understanding of knowledge that is relational and situated. Similarly, Hawaiian scholar Aluli-Meyer (2014) argues that Hawaiian epistemology has relevance beyond the locations from where it originates, its relevance is universal. What Aluli-Meyer proposes instead is a revision of the concept of universality, as emerging from specificity. Therefore she proposes a place-specific understanding of universality (Aluli-Meyer, 2014).

## Decolonizing the university

Movements and calls for decolonization first and foremost arise thanks to those who struggle against colonialism on the ground. These include Igbo Women's Wars, Lakshmi Bai—Queen of Jhansi in Northern India, thinkers such as Albert Memmi, Aimé Césaire, Franz Fanon and the rebellions of enslaved people and freedom fighters such as Toussaint L'Ouverture (Barnett-Naghshine and Pattathu, 2021). Then, during the process of political decolonization in the twentieth century, scholars began to question coloniality in relation to the institution of the university: how does the university participate in creating and perpetrating epistemic colonialism and other forms of coloniality? (Mignolo and Walsh, 2018; Santos, 2018). As Barnett-Naghshine and Pattathu (2021) note, these postcolonial scholars reversed the European gaze, and considered the conditions of knowledge production from the perspective of South America, South Asia and the Middle East (e.g., Said, Spivak, Bhabha, Lorde, Grosfoguel). In these scholars' understandings, coloniality is inextricably linked with the project of modernity, racism and global capitalism.

Importantly, this form of scholarship has received pointed critique. For instance, Cusicanqui (2013, p. 98) argues that “Mignolo and company have built a small empire within an empire,” by appropriating the contributions of subaltern scholars. Further, Tuck and Yang (2012) warn that in order not to derail decolonizing efforts, the focus should remain on the return of land and cultural resources to Indigenous guardianship.

Not only has this scholarship on decolonization been growing for some decades, but also with the #RhodesMustFall, #FeesMustFall and #BlackLivesMatter movements, decolonizing gained widespread attention within universities around the world. For instance, universities in the UK, such as the University of Aberdeen, have instituted “decolonzing the curriculum steering group.” However, since very few of these efforts attend to questions of epistemic coloniality and the relationship between ways of knowing and ways of being, a raft of pitfalls occur.

When universities take on the discourse of decolonization it often becomes a box-ticking exercise (Gopal, 2021). Many scholars join the “bandwagon of decolonization” due to the competitive academic world, and in so doing reduce the effectiveness of such terms (Moosavi, 2020). Mafeje's (2001, p. 107) critique of anthropology post-Apartheid in South Africa is an important warning in relation to the burgeoning decolonization discourse: care must be taken that those scholars intent on decolonizing their discipline don't end up as “conservative rebels” implicated in the reproduction of the academy.” Take for instance Group for Debates in Anthropological Theory (GDAT) 2022, which debated a decolonial anthropology. Despite the topic, none of the presenters questioned the hegemonic Western academic onto/epistemology they were employing. And, a conference report about the European Association of Social Anthropologists (EASA) 2022 meeting in Belfast noted how actors from the Global South were only included as tokens; that decolonization is being turned into a dominant paradigm, whereby individuals “[p]ossessing vast cultural, economic and symbolic capital (the product of epistemological extractivism from Global South sources) … become celebrities of the postcolonial canon” (Ballestero, 2022).

## The pluriversity

A key way in which the system of universities remains in collusion with coloniality, relates to the notion of the “universality” of knowledge that underpins the *uni*versity (Escobar, 2018). Similarly, the concept of universality in rights discourses has also been critiqued as perpetuating that colonial project (Ram, 2008). Such critiques are necessary and important, especially since mainstream Western science mostly continues to perpetrate epistemic colonialism. However, the way forward may not be best served by an either or logic: either adopting a human rights stance *as is*, or rejecting it entirely. The anthropologist Ram (2008) carried out fieldwork with women and advocacy groups in Bangladesh. These women embraced the notion of the universal human rights because it enabled them to form a defense against gender-based violence. What this suggests, and in many ways resonates with Aluli-Mayer's argument for a universality rooted in the specific, is a pragmatic approach, where the particularlity of circumstances needs to be taken seriously.

Another possibility is Robbins's (2010) “proposals for universals,” where different ways of knowing and being are understood as benefitting world populations and ecologies. This is in the spirit of Latour's (2004) proposal for a “New Constitution,” where all matters of fact are destabilized, acknowledged as matters of concern, but then temporarily instituted back into matters of fact until such a time when they need to return to being matters of concern. Therefore, one proposal for a universal would be a system of *pluriversities*, based on the idea of the *pluriversity* developed by Mbembe (2015). The *pluriversity* would be characterized by onto/epistemic plurality and working toward this would require a fundamental revision of how “knowledge” is understood and of the ways disciplines are constituted. The pluriversity would be built on:

- *Plurality of knowledge*: The pluriversity entails a form of pluralism which is neither relativistic nor atomising. In this definition, plurality does not lead to incommensurability and ontological separation between different ways of knowing. Rather, the pluriversity thrives on engagements between myriad distinguishable but always interweaving ways of knowing.
- *Rethinking ontology and epistemology as onto/epistemologies:* For such an understanding of plurality, a refusal to abstract epistemology from ontology is central. In order to eliminate extractive academic practices, the pluriversity will heed and further developing place-based understandings of knowledge (Watts, 2013; Aluli-Meyer, 2014).

With these reformulations of what “knowledge” is, no longer construed as a free floating “good,” and therefore a reformulation of the fundamental premises of science, it will be possible for the implementation of the right to science to contribute toward making a liveable future, where diverse, self-determining communities can flourish; communities that value connections to each other and to the environment.

## Author contributions

CG: Writing – original draft.
